# Echo-guided seldinger technique facilitates ascending aorta cannulation in type A aortic dissection

**DOI:** 10.1186/s13019-022-01939-y

**Published:** 2022-08-20

**Authors:** Yoshito Inoue

**Affiliations:** Department of Cardiovascular Surgery, Tokyo Dental College Ichikawa General Hospital, 5-11-13 Sugano, Ichikawa, Chiba 272-8513 Japan

**Keywords:** Aortic dissection, Ascending aorta cannulation, Central cannulation, Malperfusion, Type A dissection

## Abstract

**Background:**

The ascending aorta is the most simple and rapid arterial access for the establishment of antegrade systemic perfusion. In acute type A aortic dissection, prompt establishment of antegrade central perfusion, especially in unstable hemodynamic emergency operations, help to diminish organ malperfusion and to prevent retrograde embolism. The effectiveness as well as the safety of antegrade perfusion under ultrasonographic guidance through the dissected ascending aorta was evaluated for the repair of type A aortic dissection utilizing a new echo stabilizer.

**Results:**

Ascending aortic cannulation was successfully performed in 64 consecutive patients, using the Seldinger technique, with the hands-free continuous-echo monitoring, utilizing a new stabilizer. Epiaortic 2-Dimensional and color Doppler imaging provided real-time monitoring for the placement and proper perfusion of ascending aorta cannulation.

**Conlusions:**

Ascending aorta can routinely provide a rapid and reliable route of antegrade central systemic perfusion in type A acute aortic dissection. The echo-guided stabilizer-assisted cannulation method can safely provide a rapid and reliable route for antegrade central perfusion during in type A dissections repair.

**Supplementary Information:**

The online version contains supplementary material available at 10.1186/s13019-022-01939-y.

## Introduction

The prompt establishment of antegrade central perfusion, especially during hemodynamically unstable emergency operations, can reduce organ malperfusion and facilitates rapid core cooling for organ protection during type A dissection repair [1]. We refined our cannulation technique [2] by stabilizing the echo probe. The hands-free monitoring of the entire process facilitates ascending aortic cannulation.

## Methods

We performed a single-center retrospective study, for which ethical approval was waived by the Tokyo Dental University Ichikawa General Hospital Ethics Committee. Surgery was performed through a median sternotomy. The ascending aorta was cannulated using the Seldinger technique in 179 consecutive patients between 2001 and 2020. Patients were treated surgically by prosthetic graft replacement of the ascending aorta/hemiarch or total arch under deep hypothermic circulatory arrest (DHCA). Before 2009, decompression of the cannulation site via femoral artery inflow and right atrial circuit was performed before ascending aorta cannulation [1]. However, after the initial experience, we made sure that decompression by femoral cannulation is not necessary. Thus we quitted femoral incision and cannulated only ascending aorta after 2009. We reviewed 64 of these cases after 2009, when we used a stabilizer arm (mean age 67.6 ± 11.8, 36 male 28 female). Cardiopulmonary bypass (CPB) was initiated by ascending aorta inflow and right atrial outflow. Femoral artery preparation was not performed. The institutional ethics committee approved this study and waived the need for patient consent.

**Fig. 1:**
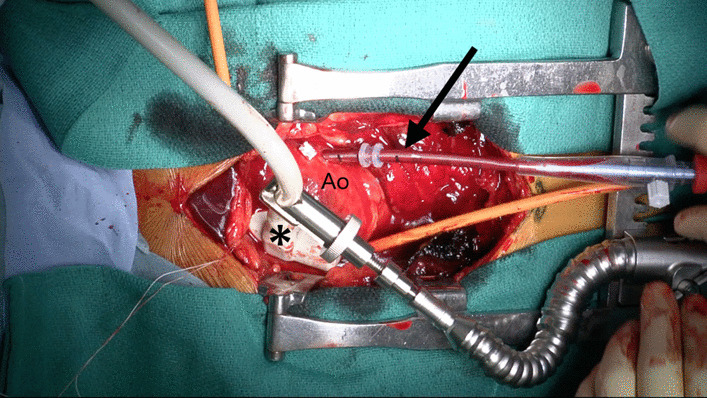
The echo probe was fixed along the ascending aorta by the stabilizer arm. Ao = aorta, asterisk = echo probe, arrow = inflow cannula

A single pledgetted 4-0 polypropylene mattress suture was placed at the left lateral side of the ascending aorta, adjacent to the pulmonary artery, for puncture. After the tip of the puncture needle was confirmed within the true lumen, a guidewire and staged dilators were inserted, step-by-step. Then, a spindle-shaped flexible cannula (18-20 Fr, Fem-Flex II arterial cannulae: Edwards Life Sciences Research Medical Midvale, Utah) was inserted along the guidewire into the true lumen of the ascending aorta (Fig. 1).

**Fig. 2:**
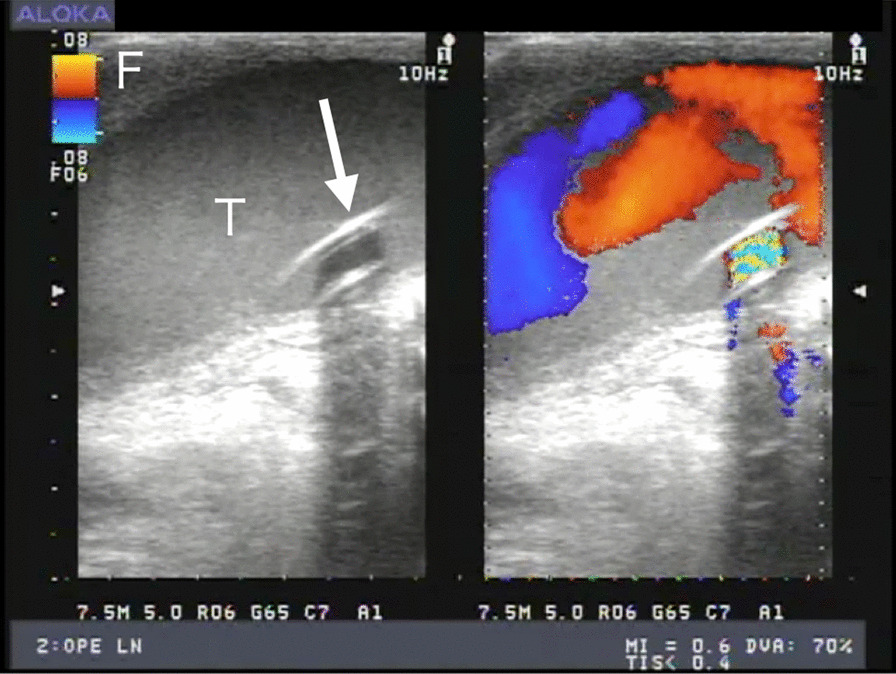
An long-axis echo image of ascending aorta cannulation. T = true lumen, F = false lumen, arrow = inflow cannula

**Fig. 3:**
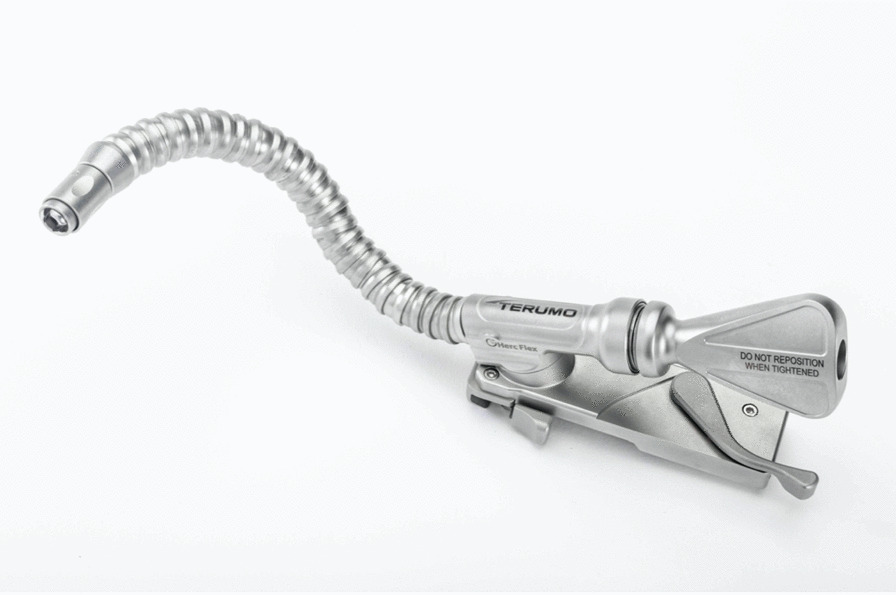
The universal stabilizer arm

**Fig. 4:**
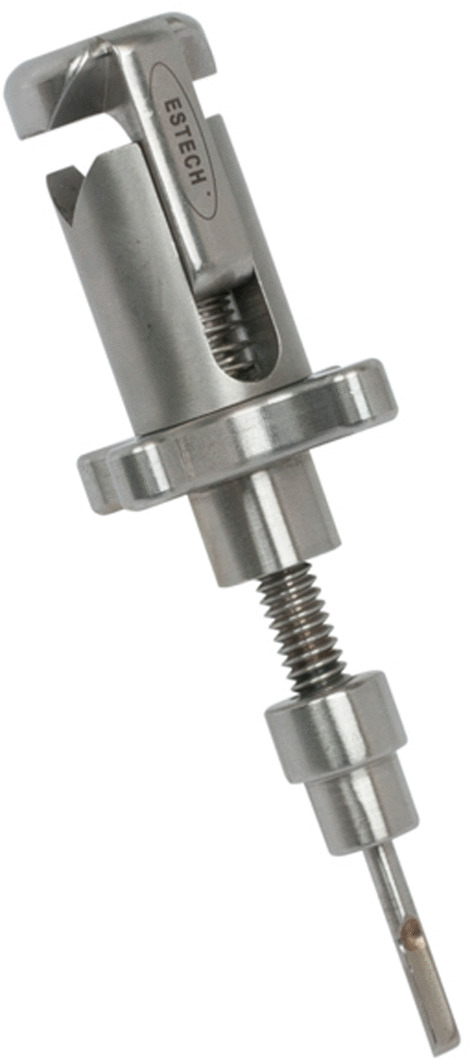
The instrument holder was attached to the stabilizer arm to fix the echo probe

The Dual-dynamic display mode epiaortic ulstrasonography was used to guide the cannulation (Fig. 1), using 2-dimensional cross-sectional and color-flow mapping (Prosound SSD-3500: Aloka Co Ltd, Tokyo). We used the new Hercules Universal Stabilizer Arm (Fig. 2; Terumo Cardiovascular Systems Corporation, Ann Arbor, Michigan) during cannulation. The T-account-form echo transducer (Linear Array Probe; UST-5534T-7.5: Aloka Co Ltd, Tokyo Japan) was held by an instrument holder (Figs. 3, 4) and was placed along the ascending aorta (Fig. 1). The proper location of the cannula and perfusion flow were confirmed within the true lumen, under real-time epiaortic echo monitoring.

## Results

Antegrade central perfusion via the ascending aorta was safely performed in all cases, with optimal perfusion pressure and sufficient pump flow. There was no conversion or disruption of the cannulation site. Antegrade central perfusion through the true lumen was easily confirmed by real-time epiaortic 2-dimensional and color Doppler echo images (Additional file 1: Video 1). The inflow cannula was directly confirmed within the true lumen in all cases. DHCA (16 ℃ at the blood temperature of the venous drainage cannula) was established within one hour of skin incision. The durations from skin incision to the intiation of CPB, from CPB to DHCA, and of DHCA were 28.4 ± 10.3, 26.5 ± 4.5, 28.5 ± 7.5 min respectively. Transesophageal echo was peroformed by the anesthesiologist, which helped to confirm true lumen expansion at descending aorta after prosthetic graft replacment.

Seven in-hospital deaths were recorded (7/61, 11.4%), caused by multi-organ failure in 3 cases, low output syndrome in 1 case, pneumonia in 1 case, and bowel ischemia in 2 cases. Five postoperative strokes (5/61, 8.2%), including two strokes that occurred later on the 6th and 14th post operative days.

## Discussion

Ascending aortic cannulation, using the Seldinger technique, in combination with stabilizer-assisted epiaortic 2-dimensional and color Doppler echocardiographic guidance, facilitated the establishment of systemic antegrade perfusion in a safe and reproducible manner. Epiaortic echo, combined with transesophageal echo imaging, ensured the accuracy of true lumen perfusion [2]. By utilizing the stabilizer arm (Figs. 3, 4), a continuous fixed-point image inside aorta contributed to successful establishment true lumen cannulation.

The stabilizer-assisted echo-guided technique has several advantages. First, it enables the hands-free continuous monitoring of the guidewire and fixed-point observation inside the aorta, which contributes to the safe and secure cannulation. Second, the aortic cannulation can be performed by a single surgeon, allowing the assistant surgeon to prepare for the next surgical steps, if necessary.

Rapid systemic cooling and the prompt cessation of the dynamic intimal flap motion caused by central perfusion may contribute to reduced organ malperfusion [2]. Recently, Shimura et al. reported that ascending aorta cannulation could potentially minimize brain malperfusion [3]. It might be because the systemic cooling, by antegrade central perfusion, can promptly induce cardiac arrest, preventing the dynamic collapse of the true lumen [3].

## Conclusions

In summary, using a stabilizer-assisted echo-guided Seldinger technique, the ascending aorta can routinely provide a rapid and reliable route for antegrade central perfusions during type A dissections repair.

## Supplementary Information

**Additional file 1.** Video 1 The actual cannulation procedures of (1) thrombosed false lumen case, and (2) perfused false lumen case.

## Data Availability

The data used during this study are available from the corresponding author on reasonable request.

## Abbreviations

DHCA: Deep hypothermic circualtory arrest
CPB: Cardiopulmonary bypass
